# Investigation of enzalutamide, docetaxel, and cabazitaxel resistance in the castration resistant prostate cancer cell line C4 using genome-wide CRISPR/Cas9 screening

**DOI:** 10.1038/s41598-023-35950-7

**Published:** 2023-06-03

**Authors:** Jakob Haldrup, Simone Weiss, Linnéa Schmidt, Karina Dalsgaard Sørensen

**Affiliations:** 1 Department of Molecular Medicine, Aarhus University Hospital, Aarhus, Denmark; 2 Department of Clinical Medicine, Aarhus University, Aarhus, Denmark

**Keywords:** Prostate cancer, High-throughput screening

## Abstract

Enzalutamide, docetaxel, and cabazitaxel treatment resistance is a major problem in metastatic castration resistant prostate cancer (mCRPC), but the underlying genetic determinants are poorly understood. To identify genes that modulate treatment response to these drugs, we performed three genome-wide CRISPR/Cas9 knockout screens in the mCRPC cell line C4. The screens identified seven candidates for enzalutamide (*BCL2L13, CEP135, E2F4, IP6K2, KDM6A, SMS,* and *XPO4*), four candidates for docetaxel (*DRG1, LMO7, NCOA2,* and *ZNF268*), and nine candidates for cabazitaxel (*ARHGAP11B, DRG1, FKBP5, FRYL, PRKAB1, RP2, SMPD2, TCEA2,* and *ZNF585B*). We generated single-gene C4 knockout clones/populations for all genes and could validate effect on treatment response for five genes (*IP6K2*, *XPO4*, *DRG1*, *PRKAB1,* and *RP2*). Altered enzalutamide response upon *IP6K2* and *XPO4* knockout was associated with deregulation of AR, mTORC1, and E2F signaling, and deregulated p53 signaling (*IP6K2* only) in C4 mCRPC cells. Our study highlights the necessity of performing individual validation of candidate hits from genome-wide CRISPR screens. Further studies are needed to assess the generalizability and translational potential of these findings.

## Introduction

Prostate cancer (PC) is the second leading cause of cancer-related death among men in the Western world^1^. While organ-confined PC is curable, advanced metastatic PC generally has a poor prognosis^2,3^. Androgen deprivation therapy constitutes the backbone for first-line treatment of advanced metastatic PC. However, resistance invariably develops, and patients enter a second phase of disease known as metastatic castration-resistant PC (mCRPC)^2,4^. Despite castrate levels of serum androgens, the androgen receptor (AR) remains a key driver of mCRPC^5^. The importance of AR signaling has prompted development of second-generation androgen signaling pathway inhibitors, including enzalutamide, that directly target AR^6^. Other approved therapeutics for mCRPC include the microtubule-stabilizing chemotherapeutics docetaxel and cabazitaxel, which exert their effects through G2M cell cycle arrest and induction of apoptosis^7^.

Unfortunately, primary and acquired resistance commonly occurs^8^. Known mechanisms of enzalutamide resistance include AR re-activation by mutations, genomic amplification, or alternative splice variants (*e.g.* AR-V7), intra-tumoral androgen synthesis, and AR-bypass via the glucocorticoid receptor^6^. Known taxane resistance mechanisms include activation of drug efflux pumps such as ABCB1^9^ and upregulation of the CCL2 chemokine^10,11^. A better understanding of the genes that contribute to treatment response in mCRPC is needed to guide more personalized treatment selection, which in the future could help improve response rates and patient survival, while also reducing side effects.

The clustered regularly interspaced short palindromic repeats (CRISPR)/Cas9 technology has emerged as a powerful tool for genome editing and high-throughput screening of the genetic factors underlying *e.g.* treatment resistance. Genome-wide libraries that consist of thousands of unique single guide RNAs (sgRNAs) can be employed to target and knockout each protein-coding gene in human cell lines. By comparing the individual sgRNA abundance between treatment vs. vehicle at a given time point, significantly enriched or depleted sgRNAs can be identified, unveiling the corresponding gene(s) that modulate cellular response towards treatment^12^.

Here, we performed three genome-wide CRISPR/Cas9 knockout screens in the mCRPC cell line C4 to identify novel genes involved in enzalutamide, docetaxel, and cabazitaxel resistance, respectively.

## Results

### Genome-wide CRISPR/Cas9 screening of enzalutamide, docetaxel, and cabazitaxel resistance

**Figure 1:**
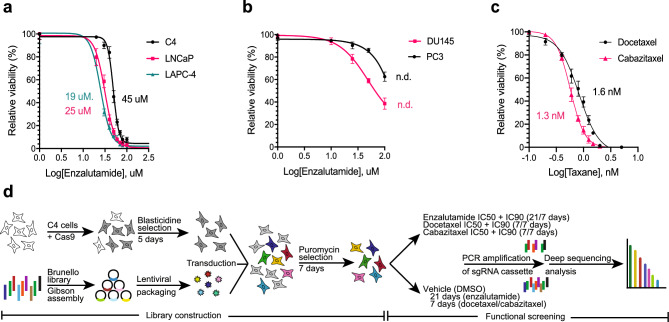
Genome-wide CRISPR/Cas9 screening of enzalutamide, docetaxel, and cabazitaxel resistance in C4 cells. (**a**, **b**) Enzalutamide dose–response curves for (**a**) C4, LNCaP, and LAPC-4 and (**b**) DU145 and PC3. (**c**) Docetaxel/cabazitaxel dose–response curves for C4. Cells were grown with drug for 3 days before alamarBlue fluorescence readout to determine viability relative to matched vehicle (DMSO) controls. N.d.: IC50 not determined. (**d**) Schematic presentation of the workflow for the genome-wide CRISPR/Cas9 screens.

To identify genes that modulate response to enzalutamide, docetaxel, or cabazitaxel treatment in PC cells, we designed three genome-wide CRISPR/Cas9 knockout screens. Initially, to identify a suitable cell line model for the AR-targeting drug enzalutamide, we performed dose–response experiments in a panel of five PC cell lines with AR expression (LAPC-4, LNCaP, and C4) or without AR expression (PC3 and DU145), respectively^13^. The three AR positive cell lines exhibited sigmoidal dose–response curves for enzalutamide, with C4 being less sensitive (IC50: 45 µM) than LNCaP (IC50: 25 µM) and LAPC-4 (IC50: 19 µM) (Fig. 1A). As expected, the two cell lines without AR expression (PC3 and DU145) were highly resistant to enzalutamide treatment and exhibited non-sigmoidal dose–response curves (Fig. 1B).

Based on this, the C4 cell line was selected for the genetic screening as it (i) showed intermediate resistance to enzalutamide and thus should be suited for identification of genes that confer increased drug resistance upon knockout (positive hits) as well as genes that confer decreased drug resistance upon knockout (negative hits), (ii) is easy to culture and expand, and (iii) is amenable to lentiviral transduction. C4 also responded to docetaxel and cabazitaxel in a dose-dependent manner (Fig. 1C), and hence was deemed a suitable model to use in the genetic screening for all three drugs.

To set up the CRISPR/Cas9 screens (Fig. 1D), C4 cells were first transduced with a Cas9-encoding vector to generate the C4_Cas9 cell line, which stably expressed Cas9 as validated by Western blotting (Supplementary Fig. S1). Subsequently, C4_Cas9 cells were transduced with the Brunello library (consisting of 1,000 non-targeting control sgRNAs and 76,441 sgRNAs targeting 19,114 genes)^14^ at an MOI of ~ 0.3, and subjected to puromycin selection for 7 days. Aliquots of the library-transduced C4_Cas9 cell population were treated with IC50 and IC90, respectively, of enzalutamide, docetaxel, or cabazitaxel for 7 or 21 days (Supplementary Table S1). An IC50-matched vehicle (DMSO)-treated control was run in parallel for each drug. At the end of selection, genomic DNA was extracted from the surviving cell pool in each sample, sgRNA regions were PCR-amplified and deep sequenced, and sequencing data was mapped to the Brunello library to obtain sgRNA read counts.

Our quality control analysis showed successful achievement of > 40 million library-mapped reads per sample, corresponding to the desired mean coverage of > 500X per sgRNA. Over 99.4% of the original sgRNA library was represented in each sample. The Gini index was low for all samples (range: 0.06–0.08), indicating homogenous sgRNA read count distribution within each sample (Supplementary Table S2). Inter-sample homogeneity was assessed by plotting the distribution of log2-normalized read counts for each sample (Supplementary Fig. S2), which revealed that median sgRNA read counts were similar between all samples. Together, this confirmed that the three screens were performed successfully.

Next, we used MAGeCK to identify top enriched and top depleted sgRNAs (Supplementary Data 1) in drug-treated samples (IC50 and IC90, respectively) vs. vehicle-treated control for all three drugs, and to rank the corresponding top candidate genes (Supplementary Data 2). These lists of top positive hits (genes whose sgRNAs were enriched in the drug-treated sample) and top negative hits (genes whose sgRNAs were depleted in the drug-treated sample) for each of the three drugs constituted the basis for our further analyses of genes that may be involved in enzalutamide, docetaxel, and/or cabazitaxel resistance in C4 mCRPC cells.

Reactome pathway analysis (Supplementary Fig. S3) revealed that top hits for enzalutamide were enriched for pathways related to gene expression and the cell cycle. In addition, for enzalutamide, top negative hits were enriched for pathways related to signal transduction and development. Of note, several of these pathways related specifically to p53, TGF-β, and E2F signaling, which have previously been linked to enzalutamide resistance^15^, thereby supporting the validity of our screen. For docetaxel, positive hits were enriched for pathways related to e.g. signal transduction, cellular response to stimuli, and the immune system. Negative hits were enriched for pathways related to p53 and TGF-β signaling, suggesting that p53 and TGF-β signaling can also mediate docetaxel response in C4 mCRPC cells. For cabazitaxel, negative hits were enriched for pathways related to gene expression, while positive hits were enriched for pathways related to chromatin organization, the cell cycle, and protein metabolism pathways (Supplementary Fig. S3), consistent with the known mechanism of action of cabazitaxel that interferes with microtubules to cause cell cycle arrest^7^.

### Selection of top hits for individual validation of screen results

**Figure 2:**
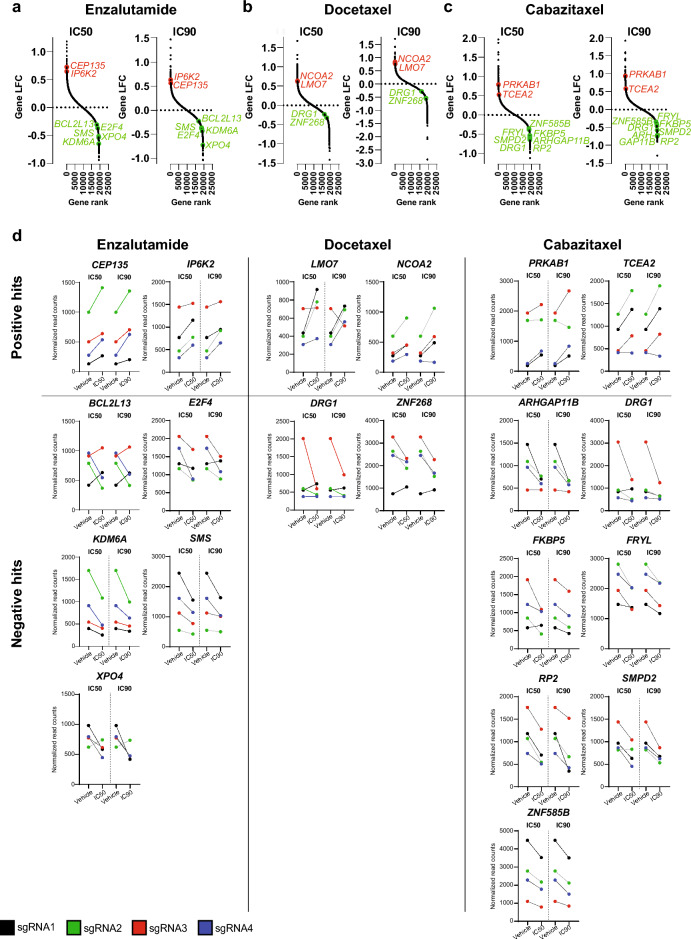
Selection of top candidate hits (genes) for individual validation. (**a**–**c**) For each plot, the y-axis shows the negative log fold change (LFC) as calculated by MAGeCK for each gene between treatment and vehicle (DMSO) conditions, while the x-axis shows the rank number for individual genes, as ranked from highest to lowest LFC determined by MAGeCK. Results from both the IC50 and the IC90 sample for the enzalutamide, docetaxel, and cabazitaxel CRISPR/Cas9 screens are presented. Genes selected for individual validation of altered treatment resistance are indicated in red for positive hits (genes whose sgRNAs were enriched in the drug-treated sample) or green for negative hits (genes whose sgRNAs were depleted in the drug-treated sample). (**d**) sgRNA read count differences between drug- and vehicle (DMSO) treated samples for all candidate genes selected for individual validation.

Next, for individual validation, we selected between four and nine top candidate positive/negative hits identified for each drug in the genome-wide screens (Fig. 2A–C). We exclusively selected candidate genes supported by at least 3 (of the 4) sgRNAs (Fig. 2D), and with a read count difference between the drug- and vehicle-treated sample of at least 250 in both the IC50 and IC90 screen, and with detectable expression (FPKM ≥ 1) in C4 cells. Thus, a total of 20 candidates were selected, including 19 unique genes (*DRG1* selected for both docetaxel and cabazitaxel): *CEP135* and *IP6K2* (enzalutamide, positive hits), *BCL2L13*, *E2F4*, *KDM6A*, *SMS*, and *XPO4* (enzalutamide, negative hits), *LMO7* and *NCOA2* (docetaxel, positive hits), *DRG1* and *ZNF268* (docetaxel, negative hits), *PRKAB1* and *TCEA2* (cabazitaxel, positive hits), and *ARHGAP11B*, *DRG1*, *FKBP5*, *FRYL*, *RP2, SMPD2,* and *ZNF585B* (cabazitaxel, negative hits) (Supplementary Table S3).

### Generation of single-gene knockout cell lines

**Figure 3:**
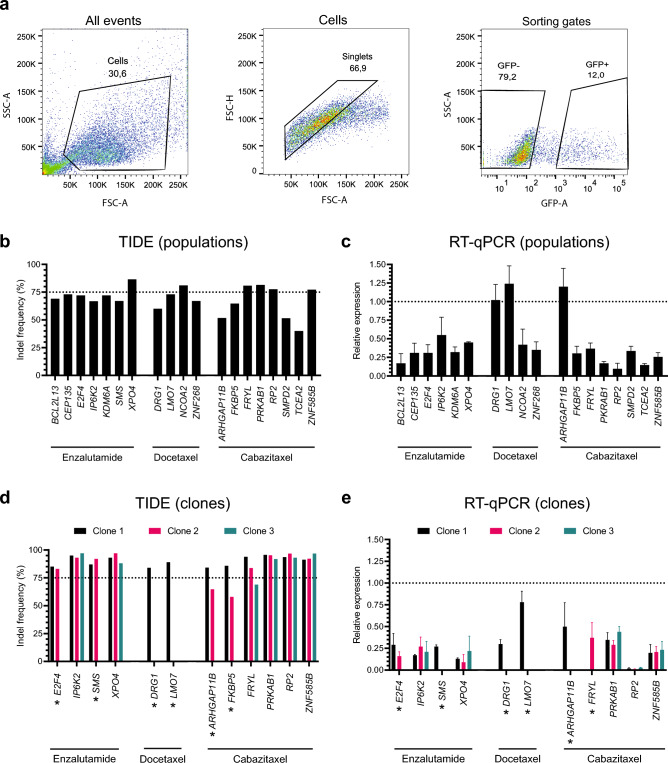
Generation of knockout populations and clones in C4_Cas9. (**a**) Gating strategy for FACS sorting used to generate knockout cell lines. The FSC-A/SSC-A plot defined the cell population for sorting, the FSC-A/FSC-H plot defined singlets, and the GFP-A/SSC-A plot defined gates for sorting into GFP-positive and GFP-negative cell populations. (**b**, **d**) TIDE indel frequencies for knockout populations (B) and knockout clones (D) in C4_Cas9 for the indicated candidate genes. (**c**, **e**) Relative expression values from RT-qPCR of candidate genes in the knockout populations (C) and knockout clones (E) as compared to wildtype control (C4_Cas9). Expression was normalized to *UBC* (enzalutamide and docetaxel candidates) or *GAPDH* (cabazitaxel candidates). Data represents one biological sample analyzed in technical triplicates, for which SD is presented by error bars. *: Less than 3 knockout clones were established.

Next, we generated single-gene knockout cell lines for each candidate by transfection of the C4_Cas9 cell line with the best-performing sgRNA (highest read count difference between vehicle and treatment in the screen) for each gene. Using FACS (Fig. 3A), we sorted transfected cells into populations (C4_pop_#GeneName) and single cell clones (C4_scc_#GeneName_#Number) to establish single-gene knockout cell lines (Supplementary Table S4). One knockout population was successfully established for each of the 19 unique candidate genes. In addition, one to three knockout clones (28 in total) were successfully established for 12 of the candidate genes (*E2F4*, *IP6K2*, *SMS*, *XPO4*, *DRG1*, *LMO7*, *ARHGAP11B*, *FKBP5*, *FRYL*, *PRKAB1, RP2*, and *ZNF585B*). Generation of single-gene knockout clones was not successful for the remaining 7 candidates (*BCL2L13*, *CEP135*, *KDM6A*, *NCOA2*, *ZNF268*, *SMPD2*, and *TCEA2*).

Genomic knockout efficiency was validated by TIDE indel frequency analysis^16^. Mean indel frequency for the 19 knockout populations was 69.1% (range: 40.0–86.5%) (Fig. 3B), while the 28 knockout clones had a higher mean indel frequency of 88.2% (range: 57.9–97.0%) (Fig. 3D). Consistent with this, mean expression level of the candidate genes, as determined by RT-qPCR, was 44.6% (range: 9.7–124.1%) in the knockout populations and 25.1% (range: 0.9–78.0%) in the knockout clones, as compared to wildtype C4_Cas9 (Fig. 3C and E). In summary, we generated 19 knockout populations (one for each unique candidate gene) and an additional 28 knockout clones for 12 of the genes (1–3 clones per gene) (Supplementary Table S4).

### Enzalutamide resistance is increased upon *IP6K2* knockout and decreased upon *XPO4* knockout, while cabazitaxel resistance is increased upon *PRKAB1* knockout and decreased upon *DRG1* and *RP2* knockout

Next, we performed 10-point dose–response assays to validate the phenotypic effect of knockout for each of the 19 candidate genes identified in the CRISPR/Cas9 screens. Cell viability after drug treatment for each single-gene knockout population/clone was determined using the alamarBlue assay and normalized to vehicle-treated controls.

**Figure 4:**
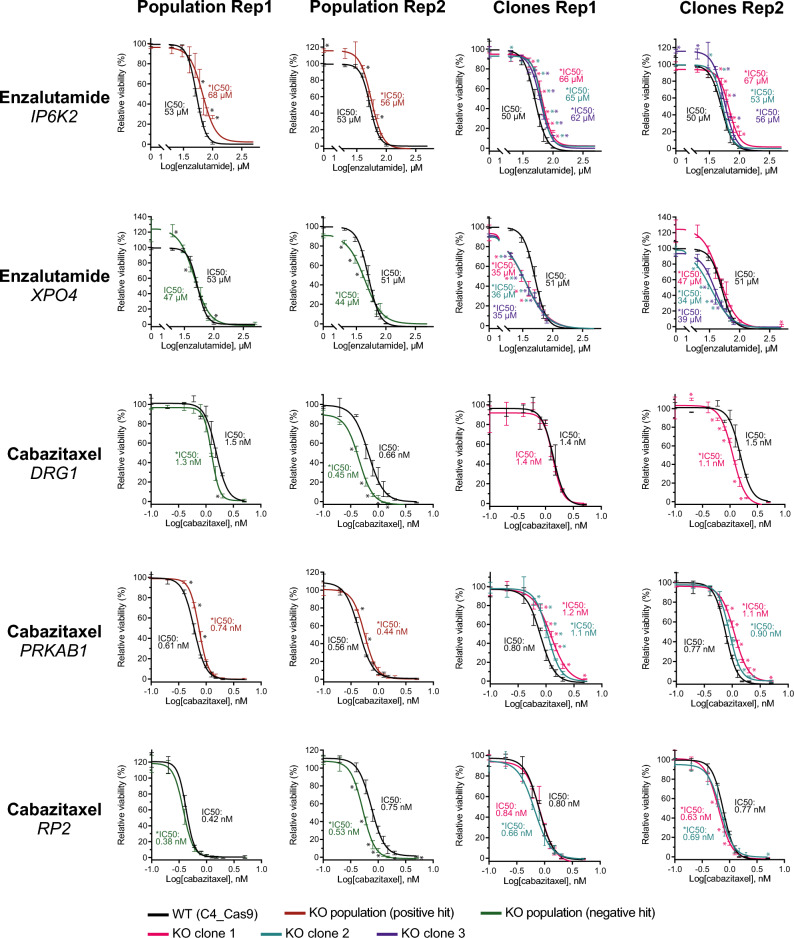
Dose–response curves for five validated candidate genes. Dose–response curves for wildtype (WT) C4_Cas9 and single-gene knockout (KO) populations (C4_pop_#GeneName) and clones (C4_scc_#GeneName_#Number) for the candidates *IP6K2, XPO4, DRG1, PRKAB1,* and *RP2.* Cell viability was determined using the alamarBlue assay after 6 days of enzalutamide treatment, respectively 3 days of cabazitaxel treatment. Dose–response curves are representative of three technical replicates. Experiments were performed in at least biological duplicates (“Rep1” and “Rep2”). Data is presented as relative viability (percentage) compared to matched vehicle-treated controls. Differences between WT and KO cells were tested for each point on the curves using an unpaired *t*-test with Holm-Sidak correction for multiple testing in GraphPad Prism. Adjusted *p*-values < 0.05 were considered statistically significant and are marked by asterisks on the figure. Differences between the IC50 of WT and knockout cell line curves were tested with an extra sum-of-squares F test in GraphPad Prism. *P*-values < 0.05 were considered statistically significant and are marked by an asterisk in front of the IC50 label on the figure.

In total, five of the candidate genes were successfully validated. For enzalutamide, *IP6K2* knockout increased resistance in C4 cells, while *XPO4* knockout decreased resistance (Fig. 4), as all knockout cell lines tested (except *XPO4* population replicate 1) showed statistically significant shifts in IC50, concordant with the screen results (Fig. 2). Similarly, for cabazitaxel, *PRKAB1* knockout increased resistance in C4 cells, while *DRG1* and *RP2* knockout decreased resistance, as all knockout cell lines tested (except *DRG1* clone 1 replicate 1 and *RP2* clone 2 replicate 1) showed statistically significant shifts in IC50 (Fig. 4).

For docetaxel, although we observed a trend towards *LMO7* knockout being associated with increased resistance in C4 cells and *DRG1* and *ZNF268* knockout with decreased resistance, in line with the screen results (Fig. 2), we could not reproducibly confirm this (Supplementary Fig. S4). We were also unable to validate the remaining candidate genes selected from the enzalutamide (*E2F4*, *KDM6A*, *SMS, CEP135,* and *BCL2L13*), docetaxel (*NCOA2*), and cabazitaxel screen (*ARHGAP11B*, *FKBP5*, *FRYL*, *SMPD2*, *TCEA2*, and *ZNF585B*) (Supplementary Fig. S5).

In summary, out of two positive and five negative hits for enzalutamide (Fig. 2B), we validated increased resistance in C4 cells upon *IP6K2* knockout (positive hit) and decreased resistance upon *XPO4* knockout (negative hit), resulting in a validation rate for enzalutamide screen hits of 2/7 (29%). None of the two positive or two negative hits for docetaxel (Fig. 2A) were validated (0/4 (0%)). Out of two positive and seven negative hits for cabazitaxel (Fig. 2C), we validated increased resistance in C4 cells upon *PRKAB1* knockout (positive hit) and decreased resistance upon *DRG1* and *RP2* knockout (negative hits), resulting in a validation rate for the cabazitaxel screen hits of 3/9 (33%).

Compared to wildtype C4_Cas9 cells, knockout populations for the five validated genes (*IP6K2, XPO4, DRG1, PRKAB1,* and *RP2*) displayed similar viability and proliferation under vehicle-treated conditions in alamarBlue (Supplementary Fig. S6) and xCELLigence assays (Supplementary Fig. S7). This indicates that the effects of *IP6K2* and *XPO4* knockout on enzalutamide resistance and of *DRG1, PRKAB1,* and *RP2* knockout on cabazitaxel resistance were not simply due to indirect effects from changes in cell viability/proliferation.

### Knockout of *IP6K2* and *XPO4* alters the transcriptome in C4 mCRPC cells

To explore the molecular pathways that mediate increased enzalutamide resistance upon *IP6K2* knockout or decreased resistance upon *XPO4* knockout in C4 cells, we treated wildtype C4_Cas9 cells, the *IP6K2* knockout clone “C4_scc_IP6K2_3”, and the *XPO4* knockout clone “C4_scc_XPO4_2” with enzalutamide at IC50 (45 µM) or matched vehicle for 6 days and performed RNA sequencing (QuantSeq; raw count data in Supplementary Data 3).

**Figure 5:**
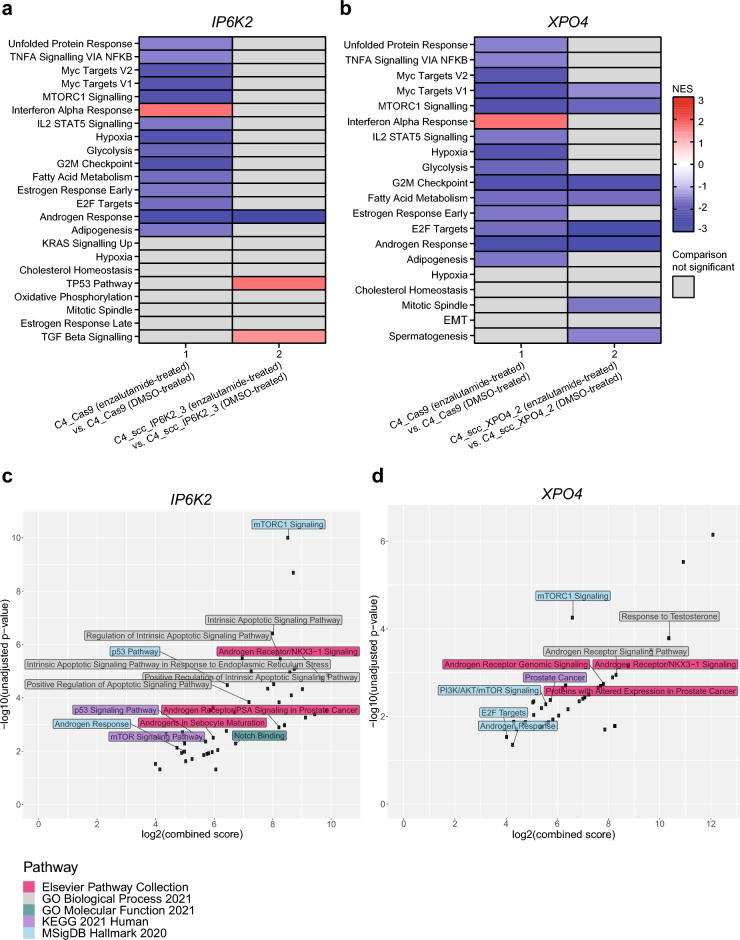
Transcriptome (QuantSeq) profiling of *IP6K2* and *XPO4* knockout clones. (**a**, **b**) GSEA analysis of the C4_Cas9 (WT), C4_scc_IP6K2_3, and C4_scc_XPO4_2 cell lines. Only significantly enriched/depleted signatures are shown (FDR < 0.05 after 1000 permutations). KO: Knockout. NES: Normalized enrichment score. (**c**, **d**) Enrichr analysis showing the top ten significant (unadjusted *p*-value < 0.05) pathways/ontologies from five different databases based on genes that were differentially expressed between vehicle (DMSO) and enzalutamide-treated C4_scc_IP6K2_3 (*n* = 90) or C4_scc_XPO4_2 (*n* = 67) cells, but not differentially expressed in C4_Cas9 cells (Supplementary Data 5). The x-axis shows the combined score (multiplied log of Fisher’s exact test *p*-value and z-score) for each pathway, as determined by Enrichr analysis^21^. The y-axis shows the unadjusted Fisher’s exact test *p*-value, as determined by Enrichr analysis^21^.

Next, using gene set enrichment analysis (GSEA)^17,18^ with log2-transformed QuantSeq CPM values as input, we investigated the pathways affected by enzalutamide (vs. vehicle) treatment in wildtype and *IP6K2* or *XPO4* knockouts, respectively. For all three cell lines, the *Androgen Response Signature* was depleted by enzalutamide treatment (Fig. 5A,B), supporting the validity of our experimental approach.

In wildtype cells, enzalutamide treatment also depleted several cell cycle-related signatures such as the *mTORC1 Signaling Signature*, the *G2M Checkpoint Signature*, and the *E2F Targets Signature* (Fig. 5A,B, column 1), while these signatures were unaffected by enzalutamide treatment in *IP6K2* knockout cells (Fig. 5A, column 2). Conversely, the *TP53 Pathway Signature* was enriched in enzalutamide-treated *IP6K2* knockout cells, but not in wildtype cells (Fig. 5A). Together, this suggests that increased enzalutamide resistance upon *IP6K2* knockout in C4 cells (Fig. 4), is mediated by cell cycle-related pathways, mTORC1 signaling and/or p53 signaling. Similarly, enrichr^19–21^ analysis of genes deregulated upon enzalutamide treatment specifically in *IP6K2* knockout cells, but not in wildtype cells (analyzed using *edgeR*^22^) identified several pathways related to mTORC1 and p53 signaling (*e.g. mTOR Signaling* and *P53 Pathway*) as well as pathways involving apoptosis and AR signaling (*e.g. Positive Regulation of Apoptotic Signaling Pathway* and *Androgen Receptor/PSA Signaling in Prostate Cancer*, respectively) (Fig. 5C and Supplementary Data 4).

GSEA results for *XPO4* knockout cells showed that enzalutamide treatment depleted the same cell cycle-related signatures as in wildtype cells (*mTORC1 Signaling*, *G2M Checkpoint*, and *E2F Targets*), although the *E2F Targets Signature* was more depleted in *XPO4* knockout cells than in wildtype cells upon enzalutamide treatment. Moreover, the *Mitotic Spindle Signature* was depleted in *XPO4* knockout cells upon enzalutamide treatment, but not in wildtype cells (Fig. 5B). Enrichr analysis of genes deregulated upon enzalutamide treatment specifically in *XPO4* knockout cells, but not in wildtype cells, supported these findings, and showed significant enrichment of pathways related to mTORC1 signaling (*e.g. mTORC1* Signaling) and E2F signaling (*e.g. E2F Targets*) in addition to pathways related to AR signaling (*e.g. Androgen Receptor Signaling Pathway*) (Fig. 5D and Supplementary Data 4). Together, this suggests that decreased enzalutamide resistance in C4 cells upon *XPO4* knockout (Fig. 4) may be mediated through augmentation of the enzalutamide-induced downregulation of cell cycle-related pathways also observed in wildtype cells (Fig. 5B, column 1).

In summary, transcriptome analyses of *IP6K2* and *XPO4* single-gene knockout clones revealed altered expression of genes related to AR, mTORC1, and E2F signaling, as well as p53 signaling (*IP6K2* knockouts only), suggesting that these pathways mediate the changes in enzalutamide resistance observed in C4 cells upon *IP6K2* and *XPO4* knockout, respectively.

## Discussion

This study is the first to report results from in vitro genome-wide CRISPR/Cas9 knockout screening of genes involved in resistance towards enzalutamide, docetaxel, and cabazitaxel in PC cells. Using the cell line C4 as a model for mCRPC, we identified and independently validated the association of two genes with enzalutamide resistance (*IP6K2* and *XPO4*) and three genes with cabazitaxel resistance (*DRG1*, *PRKAB1*, and *RP2*). Neither of these five genes have been associated with enzalutamide or cabazitaxel resistance before. We also sought to validate altered cellular drug resistance upon knockout of five other candidate genes identified from the enzalutamide screen (*BCL2L13, CEP135, E2F4, KDM6A,* and *SMS*), four candidate genes from the docetaxel screen (*DRG1, LMO7, NCOA2,* and *ZNF268*), and six other candidate genes from the cabazitaxel screen (*ARHGAP11B, FKBP5, FRYL, SMPD2, TCEA2,* and *ZNF585B*). However, none of these 15 candidate genes were validated, resulting in an overall validation rate of 5/20 (25%). This highlights the necessity of performing individual validation of candidate hits from genome-wide CRISPR/Cas9 knockout screens. While further studies are warranted and should assess the generalizability and translational potential of our findings, we propose that the extended gene hit lists from our three genome-wide screens (Supplementary Data 2) may serve as a starting point for other researchers wishing to explore the genetic basis of enzalutamide, docetaxel, and cabazitaxel resistance in PC and beyond.

In the current study, we validated that *IP6K2* knockout increased enzalutamide resistance in C4 cells (Fig. 4). A known cellular function of *IP6K2* is the generation of inositol phosphate, IP7, that is a messenger molecule involved in ATP production and regulation of cell growth^23^. While there are no previous reports about *IP6K2* in relation to enzalutamide resistance specifically or PC in general, other studies have demonstrated a pro-apoptotic role of *IP6K2* in ovarian carcinoma cells^24^ and colon cancer cells^25^, mediated by p53-binding^26^. This is in line with the deregulation of p53- and apoptosis-related pathways, which we observed in C4 mCRPC cells with *IP6K2* knockout in our transcriptome analyses.

In this study, we also validated that *XPO4* knockout decreased enzalutamide resistance in C4 cells (Fig. 4). The *XPO4* gene encodes a protein transporter that mediates nuclear import of the SOX2 transcription factor and nuclear export of the SMAD3 transcription factor^27^. *XPO4* has not been directly linked to PC or enzalutamide resistance before, but previous reports have shown that *SOX2* facilitates enzalutamide resistance in *TP53-* and *RB1-*deficient LNCaP PC cells by promoting lineage plasticity^28^. Furthermore, knockdown of *SMAD3* expression has been shown to reduce cell viability in an enzalutamide-resistant LNCaP derivative^29^, concordant with our observations in this study, where *XPO4* knockout in C4 mCRPC cells reduced viability during enzalutamide treatment.

Moreover, in the present study, we validated that *DRG1* knockout decreased cabazitaxel resistance in C4 cells (Fig. 4). *DRG1* has not previously been linked to taxane resistance, but it is known to hold tumor suppressor functions in both prostate and breast cancer^30^, where it promotes degradation of the pro-apoptotic protein Bim^31^. This might explain why *DRG1* knockout sensitized C4 cells to cabazitaxel, as decreased degradation of a pro-apoptotic protein may enhance the taxane-induced apoptosis. In our study, *DRG1* was selected as a candidate gene in both the docetaxel and cabazitaxel screen. Although we were not able to validate *DRG1* as a candidate gene for docetaxel resistance in C4 cells, we did observe a trend towards decreased docetaxel resistance upon *DRG1* knockout (Supplementary Fig. S4).

Furthermore, our results showed that *PRKAB1* knockout increased cabazitaxel resistance in C4 cells (Fig. 4). *PRKAB1* encodes the β1 subunit of AMP-activated protein kinase (AMPK), which responds to ATP depletion upon cellular stress by increasing ATP production or inhibiting ATP consumption^32^. *PRKAB1* has not previously been associated with taxane resistance, and it is currently unclear whether *PRKAB1* has an oncogenic or tumor suppressor effect in PC, as previous studies have shown contrasting results. For example, one study reported that *PRKAB1* is overexpressed in metastatic vs. primary PC, indicating an oncogenic function, whereas another study showed that deletion of the *PRKAB1* gene in mouse models conferred earlier prostate adenocarcinoma development, indicating a tumor suppressor function^33,34^. Our results are predominantly in line with a tumor suppressor function in C4 mCRPC cells, as we showed that *PRKAB1* knockout increased cabazitaxel resistance.

Finally, we validated that *RP2* knockout was associated with decreased cabazitaxel resistance in C4 cells (Fig. 4). The function of *RP2* is not yet fully elucidated and the gene has neither been implicated in PC nor taxane resistance before. However, RP2 has similarity with tubulin cofactor C and can stimulate tubulin GTPase activity, which is essential for microtubule dynamics^35,36^. Since cabazitaxel works by interfering with microtubule dynamics^7^, this may at least in part explain why *RP2* knockout sensitized C4 cells to cabazitaxel in our study.

In summary, this study identified two novel genes implicated in enzalutamide resistance (*IP6K2* and *XPO4*) and three novel genes implicated in cabazitaxel resistance (*DRG1*, *PRKAB1*, and *RP2*) in C4 mCRPC cells.

A limitation of this study is that the candidate genes were validated in only one mCRPC model (the C4 cell line). Accordingly, future studies should include validation in additional mCRPC models, such as other PC cell lines. For further in-depth analyses, cell line studies may include both 2D and 3D culture experiments as well as xenograft studies in mice. Future studies may also investigate the association between the five validated candidate genes and drug response in mCRPC patient-derived organoids.

While such further studies are warranted, they are considered to be beyond the scope of the current study, a main strength of which is that it is the first report of genome-wide CRISPR/Cas9 knockout screens for genes involved in enzalutamide, docetaxel, and cabazitaxel resistance in PC cells. As such, this report also provides the scientific community with extended gene lists of positive and negative hits from the three screens (Supplementary Data 2), which may be valuable for other researchers studying the genetic basis of enzalutamide, docetaxel and/or cabazitaxel resistance in PC and/or other cancer types.

## Methods

### Cell lines

C4, LNCaP, PC3, and DU145 cells were propagated in RPMI-1640 medium with L-glutamine (Invitrogen) supplemented with 10% heat-inactivated fetal bovine serum (Gibco) and 100 U/mL penicillin and 100 μg/mL streptomycin (RPMI 1640 + / +) (Life Technologies) at 37 °C in humidified atmospheric air with 5% CO_2_. LAPC-4 cells were propagated in RPMI 1640 + / + and stimulated with 10 nM R1881 (Sigma-Aldrich). All cell lines were authenticated by short tandem repeat (STR) profiling (IdentiCell) and repeatedly tested negative for mycoplasma (PCR Mycoplasma Detection Set, Takara Bio Inc.).

### Genome-wide CRISPR/Cas9 screening

CRISPR/Cas9 screening was performed as described previously^37^, with minor modifications. In brief, C4 cells were transduced with LentiCas9-Blast (Addgene ID: 52,962) at a multiplicity of infection (MOI) ~ 1.0 and selected by blastidicine (Sigma-Aldrich) for 5 days. The resulting C4_Cas9 population, with stable Cas9 expression, was transduced with the Brunello library^14^ (Addgene ID: 73,178) at an MOI ~ 0.3 and selected by puromycin (Sigma-Aldrich) for 7 days. Library-transduced cells were expanded and split into several arms that were exposed to, respectively, IC50 and IC90 of enzalutamide (MedChemExpress, cat. no. HY-70002), docetaxel (MedChemExpress, cat. no. HY-B0011), or cabazitaxel (MedChemExpress, cat. no. HY-15459) treatment for 7 or 21 days depending on toxicity (Supplementary Table S1). An IC50-matched vehicle (DMSO) control was analyzed in parallel for each drug. In all cases, cells were passaged every 3–4 days and a minimum of 40 million cells were re-seeded each time to maintain > 500X coverage per sgRNA. Following selection, surviving cells were harvested, genomic DNA extracted, and sgRNA abundance quantified by deep targeted sequencing as described previously^37^. The resulting FastQ files were processed by Model-based Analysis of Genome-wide CRISPR/Cas9 Knockout (MAGeCK) v. 0.5.9.2^38^ for sgRNA read mapping, normalization, and ranking (Supplementary Data 1 and Supplementary Data 2). In addition, the log fold change (LFC) of individual sgRNAs was determined using the normalized MAGeCK output.

### Reactome pathway analysis

The overlap between top 1000-ranked hits (genes) in the IC50 and IC90 screen for each drug (enzalutamide, docetaxel, and cabazitaxel) was subjected to Reactome pathway analysis^39^. Default settings were used and entities with *p*-values < 0.05 were considered significant.

### Generation of single-gene knockout cell lines

To establish individual knockout cell line populations (pop) and single cell clones (scc) for all 19 candidate genes identified in the screens (Supplementary Table S3), the best-performing sgRNA for each gene (defined as the sgRNA having the highest read count difference between vehicle vs. drug treatment in the screen; Supplementary Table S4) was cloned by a digestion/ligation reaction into the PX458 plasmid (Addgene ID: 48,138): 100 ng PX458 plasmid, 1 µL sgRNA, 1 µL BbsI Fast Digest (Thermo Scientific), 1 µL T4 DNA ligase (Thermo Fisher Scientific), 2 µL T4 DNA ligase buffer (Thermo Fisher Scientific), and H_2_O to 20 µL. The reaction was incubated in a thermocycler under the following conditions: 10 cycles of (5 min at 37 °C then 10 min at 23 °C), hold for 30 min at 37 °C, hold for 15 min at 75 °C, and store at 4 °C. The product was chemically transformed into XL2-Blue ultra-competent bacteria (AH diagnostics) and seeded onto LB agar plates containing 100 µg/mL of ampicillin. Bacterial clones were checked for correct sgRNA insert by Sanger sequencing using a primer located in the U6 promoter region of the PX458 plasmid (Supplementary Table S5). Subsequently, validated bacterial clones were grown in ampicillin-supplemented LB medium overnight before plasmid DNA purification with the NucleoBond Xtra Midi purification kit (Macherey–Nagel).

For each candidate gene, C4_Cas9 (wildtype control) cells were then transfected with the corresponding cloned PX458 plasmid using FuGENE HD Transfection Reagent (Promega). After 72 h, GFP-positive cells were isolated by FACS and either seeded as single cells directly into 96-well plates containing 200 µL conditioned RPMI 1640 + / + medium to establish single cell clones, or sorted as populations into 50 µL FBS in Eppendorf tubes and later transferred to flasks for cultivation. Following FACS, all cells were tested negative for mycoplasma.

### Validation of genomic knockout

Genomic DNA was harvested from C4_Cas9 and all knockout cell lines using the NucleoSpin Tissue Kit (Macherey–Nagel) according to the manufacturer’s instructions. Subsequently, candidate gene genomic knockout was validated by Sanger sequencing of the DNA (primer sequences listed in Supplementary Table S6) followed by TIDE indel frequency and spectrum analysis, comparing C4_Cas9 and each knockout cell line^16^.

### RT-qPCR

C4_Cas9 and knockout populations and clones were harvested into cell pellets, lysed in RLT buffer with 1% β-mercaptoethanol (Qiagen), and total RNA was purified using the RNeasy mini kit (Qiagen). cDNA was synthesized using the SuperScript II reverse transcriptase kit (Life Technologies). RT–qPCR was performed using SYBR Green PCR Master Mix (Life Technologies) (primer sequences listed in Supplementary Table S6). All RT-qPCR experiments were performed in technical triplicates and run on the ViiA™ 7 Real-Time PCR system (Applied Biosystems). QuantStudio (Applied Biosystems) was used to analyze the data. Assay specificity was validated by melting curve analysis. The mean quantity of each candidate gene was determined using the standard curve approach. *UBC* and *GAPDH* were used for normalization.

### Dose–response assays

Enzalutamide, docetaxel, and cabazitaxel were diluted in DMSO to 10 mM stocks, aliquoted, and stored at -20 °C until use. C4, LNCaP, LAPC-4, PC3, DU145, C4_Cas9, and knockout populations and clones were seeded at a density of 5000–8000 cells/well in 96-well plates coated with poly-L-Lysine (Sigma-Aldrich) and incubated for 24 h. A 10-point drug dilution series was added to the cells and incubated for 3 days (enzalutamide: C4, LNCaP, LAPC-4, PC3, and DU145), 6 days (enzalutamide: C4_Cas9 and knockout cell lines) or 3 days (docetaxel and cabazitaxel: C4_Cas9 and knockout cell lines), respectively. Cell viability was determined by incubation with 10% alamarBlue Cell Viability Reagent (Thermo Fisher Scientific) for 4 h, followed by fluorescence readout at 530 nm/620 nm using the Synergy HT reader (BioTek). Results were normalized to matched vehicle controls and presented as percent viability using GraphPad Prism v. 8.3.1. All experiments were performed in technical triplicates and repeated at least twice.

### Cell viability

Viability of untreated cells was determined for C4_Cas9 and the knockout populations C4_pop_IP6K2, C4_pop_XPO4, C4_pop_DRG1, C4_pop_PRKAB1, and C4_pop_RP2. In brief, cells were seeded at a density of 6000–8000 cells/well in 96-well plates and incubated for 72–96 h. Cell viability was determined using the alamarBlue Cell Viability Reagent, as described above. All experiments were performed in technical triplicates and repeated at least twice.

### Real-time cell proliferation assays

C4_Cas9 and the knockout populations C4_pop_PRKAB1 and C4_pop_RP2 were seeded at a density of 7000–8000 cells/well in ePlate16 wells (ACEA Bioscience). Cell proliferation was measured using the xCELLigence Real-Time Cell Analyzer (RTCA, Roche Diagnostics) with cell indices recorded every 15 min for 144 h. All experiments were performed in technical triplicates and repeated twice.

### QuantSeq whole-transcriptome analysis

C4_Cas9 and the knockout clones C4_scc_IP6K2_3 and C4_scc_XPO4_2 were seeded in TC-25 flasks and incubated for 24 h in duplicates. Cells were exposed to enzalutamide (IC50) treatment (Supplementary Table S1) for 6 days. A matched vehicle (DMSO) control was used as reference. Surviving cells were lysed directly in the flasks using RLT buffer and β-mercaptoethanol and total RNA was purified using the RNeasy mini kit (Qiagen). QuantSeq libraries (Lexogen) were generated using 500 ng rRNA-depleted total RNA, following 12 cycles of PCR. Indexed libraries were quantified using Qubit (Thermo Fisher Scientific) and fragment lengths were estimated using the Tapestation 4200 system (Agilent Technologies). Indexed libraries were single-end sequenced (75 + 1 + 6 bp) on Illumina NextSeq 500 to an average of 5 million reads/sample. Raw reads were processed to FastQ format and de-multiplexed using bcl2fastq v. 2.20 (Illumina). Adapters were discarded using trim galore v. 0.4.1. Reads were mapped to the human genome (hg19) using TopHat2 (v. 2.1.1), Bowtie2 (v. 2.1), Cufflinks (v. 2.1.1), and HTseq (v. 0.11.2) to estimate transcript abundance (read counts).

Gene set enrichment analysis (GSEA) was performed in the JavaGSEA Desktop Application (v. 4.0) using the Hallmark MSigDB collection. Log2-transformed counts per million (CPM) values were used as input. Gene sets with FDR < 0.05 after 1000 permutations were considered significant. GraphPad Prism (v. 8.3.1) was used to present the normalized enrichment score (NES) of deregulated gene sets.

The *edgeR* (v. 3.34.1)^22^ R (v. 4.1.0) package was used for differential gene expression analysis of CPM values. Genes that were differentially expressed in enzalutamide- as compared to vehicle-treated C4_scc_IP6K2_3 and C4_scc_XPO4_2 cells, but not in enzalutamide- as compared to vehicle-treated C4_Cas9 cells (Supplementary Data 5) were subsequently subjected to Enrichr Pathway analysis^19–21^. Top 10 significantly enriched (unadjusted *p*-values < 0.05) pathways/ontologies were extracted from five databases: KEGG 2021 Human, Elsevier Pathway Collection, MSigDB Hallmark 2020, GO Biological Process 2021, and GO Molecular Function 2021.

## Supplementary Information

Supplementary Information 1.

Supplementary Information 2.

Supplementary Information 3.

Supplementary Information 4.

Supplementary Information 5.

Supplementary Information 6.

## Data Availability

All data generated or analyzed during this study are included in this published article and its supplementary information files.
