# Supplementary material for: Investigation of enzalutamide, docetaxel, and cabazitaxel resistance in the castration resistant prostate cancer cell line C4 using genome-wide CRISPR/Cas9 screening
Source: Sci Rep. 2023 Jun 3;13:9043. doi: 10.1038/s41598-023-35950-7 (PMC10239467; doi:10.1038/s41598-023-35950-7)
Supplement: Supplementary file 6 — Supplementary Information 6. [file 41598_2023_35950_MOESM6_ESM.pdf]

Supplementary Information

Investigation of Enzalutamide, Docetaxel, and Cabazitaxel Resistance in the Castration Resistant Prostate Cancer Cell Line C4 using Genome-Wide CRISPR/Cas9 Screening

Authors

Jakob Haldrup[#1,2], Simone Weiss[#1,2], Linnéa Schmidt[1,2], and Karina Dalsgaard Sørensen*[1,2]

Affiliations

[1]Department of Molecular Medicine (MOMA), Aarhus University Hospital, Aarhus, Denmark
[2]Department of Clinical Medicine, Aarhus University, Aarhus, Denmark
[#]These authors contributed equally
*Corresponding author

Figures

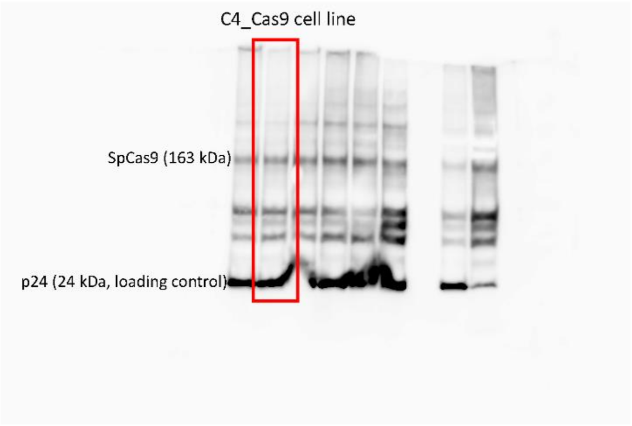

Supplementary Figure S1. Validation of Cas9 expression. Western blot confirming Cas9 expression in the C4_Cas9 cell line. The p24 protein was used as a loading control.

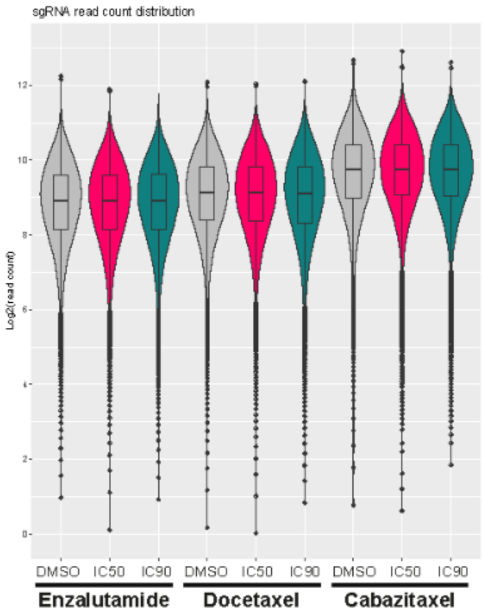

Supplementary Figure S2. Distribution of log2-transformed sgRNA read counts in each sample (IC50, IC90 and matched vehicle-treated control) for the three drugs.

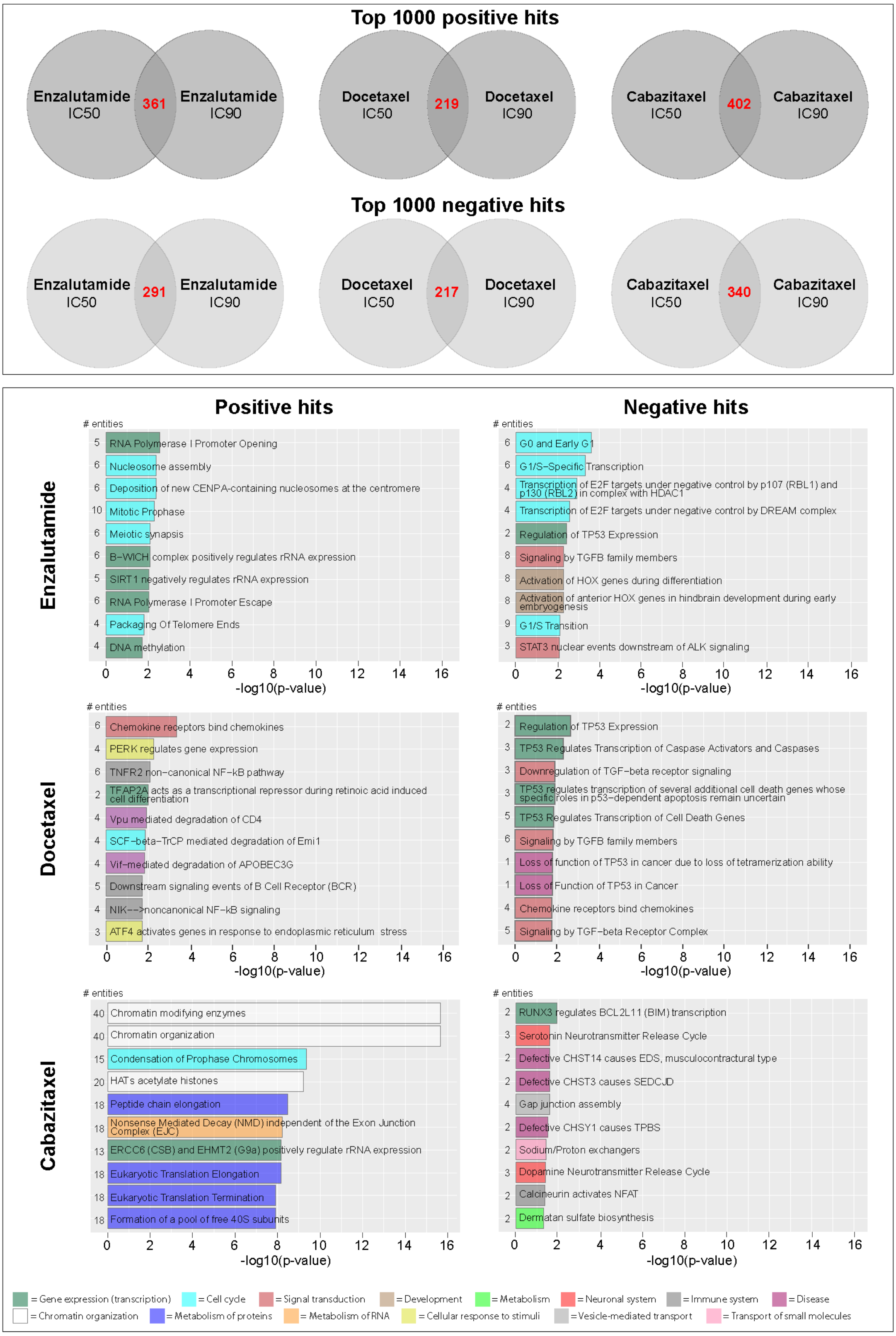

Supplementary Figure S3. Pathway analyses of top hits identified in the CRISPR screens. Upper panel shows the overlap between MAGeCK top 1000-ranked positive/negative IC50 and IC90 hits for the enzalutamide, docetaxel, and cabazitaxel screens. The lower panel shows top 10 results from Reactome pathway analysis of the overlapping hits marked with red in the upper panel. The x-axis shows uncorrected *p*-values and at the left of each top ranked pathway is noted how many entities (genes) supported the pathway.

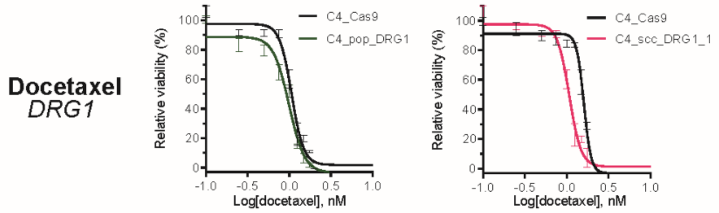

	Population	Clone 1
Control_Rep1	1.27 nM	1.60 nM
DRG1_Rep1	0.94 nM	1.07 nM

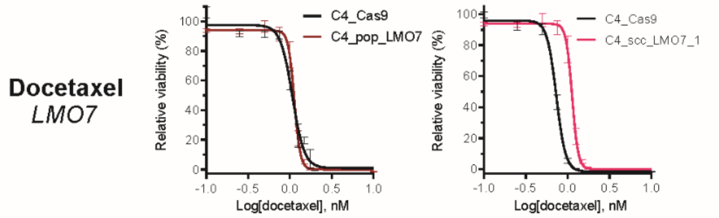

	Population	Clone 1
Control_Rep1	1.07 nM	0.73 nM
LMO7_Rep1	1.13 nM	1.13 nM

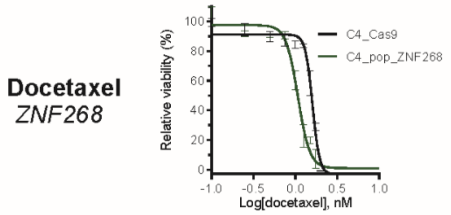

	Population	Clone 1
Control_Rep1	1.61 nM	*NA*
ZNF268_Rep1	1.07 nM	*NA*

Supplementary Figure S4. Knockout cell lines with inconclusive phenotypes. All available dose-response curves for candidate genes that were never fully validated or failed. IC50 values are listed in the tables on the right side.

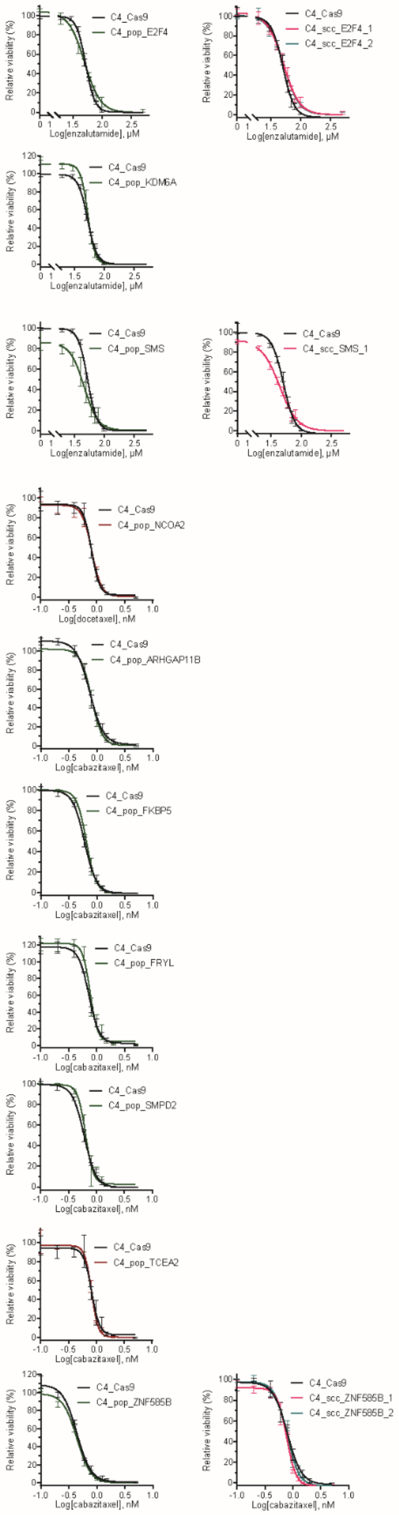

Enzalutamide
E2F4

	Population	Clone 1	Clone 2
Control_Rep1	53 μM	51 μM	51 μM
E2F4_Rep1	50 μM	51 μM	52 μM

Enzalutamide
KDM6A

	Population	Clone 1	Clone 2
Control_Rep1	53 μM	*NA*	*NA*
KDM6A_Rep1	53 μM	*NA*	*NA*

Enzalutamide
SMS

	Population	Clone 1	Clone 2
Control_Rep1	53 μM	51 μM	*NA*
SMS_Rep1	46 μM	45 μM	*NA*

Docetaxel
NCOA2

	Population	Clone 1	Clone 2
Control_Rep1	0.81 nM	*NA*	*NA*
NCOA2_Rep1	0.82 nM	*NA*	*NA*

Cabazitaxel
ARHGAP11B

	Population	Clone 1	Clone 2
Control_Rep1	0.75 nM	*NA*	*NA*
ARHGAP11B_Rep1	0.80 nM	*NA*	*NA*

Cabazitaxel
FKBP5

	Population	Clone 1	Clone 2
Control_Rep1	0.61 nM	*NA*	*NA*
FKBP5_Rep1	0.65 nM	*NA*	*NA*

Cabazitaxel
FRYL

	Population	Clone 1	Clone 2
Control_Rep1	0.73 nM	*NA*	*NA*
FRYL_Rep1	0.77 nM	*NA*	*NA*

Cabazitaxel
SMPD2

	Population	Clone 1	Clone 2
Control_Rep1	0.61 nM	*NA*	*NA*
SMPD2_Rep1	0.64 nM	*NA*	*NA*

Cabazitaxel
TCEA2

	Population	Clone 1	Clone 2
Control_Rep1	0.82 nM	*NA*	*NA*
TCEA2_Rep1	0.82 nM	*NA*	*NA*

Cabazitaxel
ZNF585B

	Population	Clone 1	Clone 2
Control_Rep1	0.44 nM	0.80 nM	0.80 nM
ZNF585B_Rep1	0.44 nM	0.77 nM	0.78 nM

Supplementary Figure S5. Knockout cell lines that failed validation. One representative dose-response curve is shown for each gene that failed validation. IC50 values are listed in the tables on the right side. For the two remaining enzalutamide hits (*CEP135* and *BCL2L13*), we did not obtain reliable dose-response data and their possible effects on enzalutamide resistance therefore remain unvalidated.

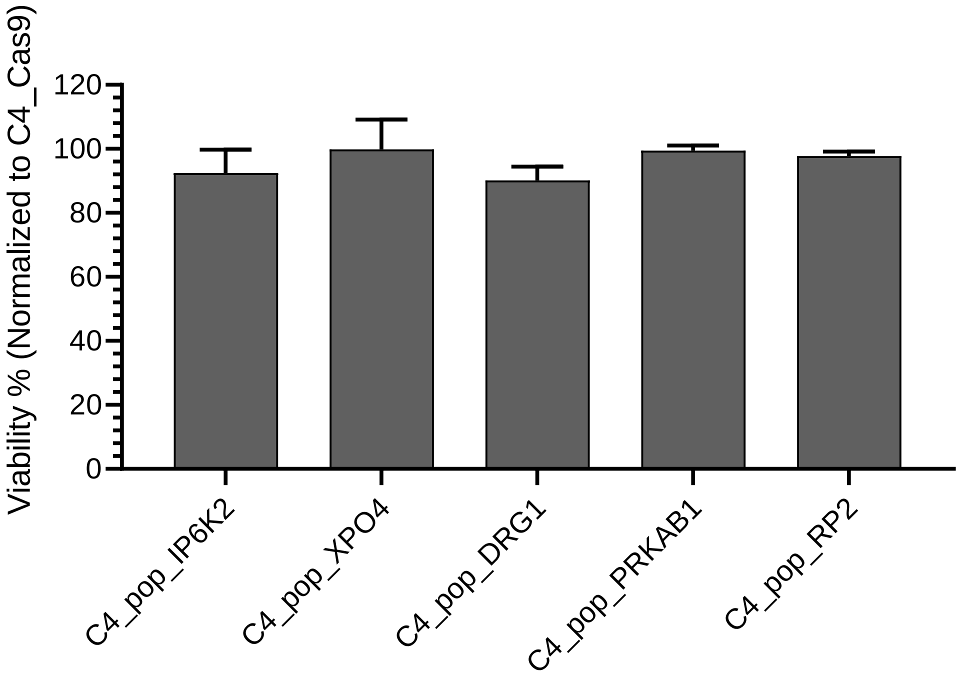

Supplementary Figure S6. Baseline (untreated) viability. Viability of the C4_pop_IP6K2, C4_pop_XPO4, C4_pop_DRG1, C4_pop_PRKAB1, and C4_pop_RP2 knockout populations treated with vehicle (DMSO) and normalized to wildtype C4_Cas9.

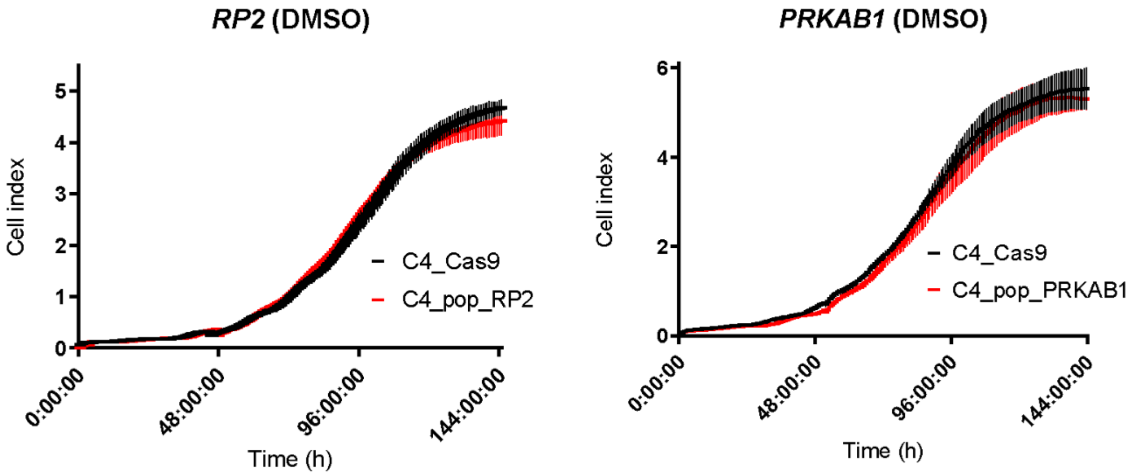

Supplementary Figure S7. Baseline (untreated) proliferation. xCELLigence experiments showing proliferation of the C4_pop_RP2 and C4_pop_PRKAB1 cell lines under vehicle (DMSO) treatment. Experiments were performed in technical duplicates and repeated twice; a representative example is shown.

Tables

Drug	IC50	IC90	Days (IC50/IC90)
Enzalutamide	45 µM	70 µM	21/7
Docetaxel	1.3 nM	2.1 nM	7/7
Cabazitaxel	1.3 nM	2.1 nM	7/7

Supplementary Table S1. Genome-wide CRISPR/Cas9 screening conditions.

Enzalutamide	Reads	Mapped	% mapped reads	Total sgRNA	#ZeroCounts	Gini Index
**IC50**	4.77E+07	4.20E+07	88	77441	385	0.08
**IC90**	5.38E+07	4.78E+07	89	77441	386	0.08
**Vehicle**	5.24E+07	4.61E+07	88	77441	364	0.08

Docetaxel	Reads	Mapped	% mapped reads	Total sgRNA	#ZeroCounts	Gini Index
**IC50**	5.82E+07	5.18E+07	89	77441	390	0.07
**IC90**	6.63E+07	5.87E+07	89	77441	433	0.08
**Vehicle**	5.22E+07	4.65E+07	89	77441	424	0.07

Cabazitaxel	Reads	Mapped	% mapped reads	Total sgRNA	#ZeroCounts	Gini Index
**IC50**	1.16E+08	1.03E+08	89	77441	322	0.06
**IC90**	9.94E+07	8.86E+07	89	77441	317	0.06
**Vehicle**	5.22E+07	4.65E+07	89	77441	424	0.07

Supplementary Table S2. Quality control information for the enzalutamide, docetaxel, and cabazitaxel screens.

Gene	Hit type	Screen		MAGeCK *p*-value	MAGeCK FDR	MAGeCK rank	MAGeCK LFC
BCL2L13	Negative	Enzalutamide	IC50:	0.00016592	0.786561	4	-0.30897
			IC90:	0.0025292	0.707595	63	-0.22729
CEP135	Positive	Enzalutamide	IC50:	0.00099322	0.620996	28	0.73037
			IC90:	0.0006578	0.643381	16	0.56489
E2F4	Negative	Enzalutamide	IC50:	0.0012652	0.786561	29	-0.37098
			IC90:	0.002499	0.707595	62	-0.42989
IP6K2	Positive	Enzalutamide	IC50:	0.00045174	0.620996	11	0.65078
			IC90:	0.00066854	0.643381	17	0.63105
KDM6A	Negative	Enzalutamide	IC50:	0.00011888	0.786561	2	-0.65202

			IC90:	0.011799	0.886703	252	-0.39001
SMS	Negative	Enzalutamide	IC50:	0.00015365	0.786561	3	-0.51718
			IC90:	0.00023955	0.579827	8	-0.36457
XPO4	Negative	Enzalutamide	IC50:	0.00043896	0.786561	9	-0.53848
			IC90:	4.9853e-05	0.487624	1	-0.72453
*DRG1**	Negative	Docetaxel	IC50:	0.00011786	0.931243	1	-0.23241
			IC90:	0.0022536	0.824488	47	-0.28438
LMO7	Positive	Docetaxel	IC50:	0.0022362	0.776828	54	0.6202
			IC90:	0.002315	0.835702	48	0.76865
NCOA2	Positive	Docetaxel	IC50:	0.0013741	0.776828	28	0.63161
			IC90:	0.0035017	0.835702	79	0.83252
ZNF268	Negative	Docetaxel	IC50:	0.0005857	0.931243	9	-0.32148
			IC90:	1.1504e-05	0.222772	1	-0.54184
ARHGAP11B	Negative	Cabazitaxel	IC50:	0.0039172	0.810049	84	-0.59851
			IC90:	0.00032041	0.837155	7	-0.72343
*DRG1**	Negative	Cabazitaxel	IC50:	0.00031575	0.726623	7	-0.61218
			IC90:	0.00010491	0.668317	3	-0.45709
FKBP5	Negative	Cabazitaxel	IC50:	0.0015814	0.810049	33	-0.52817
			IC90:	0.0022429	0.837155	47	-0.43781
FRYL	Negative	Cabazitaxel	IC50:	0.0053227	0.810049	117	-0.38204
			IC90:	0.0047904	0.842384	105	-0.34498
PRKAB1	Positive	Cabazitaxel	IC50:	0.00039657	0.269205	28	0.79049
			IC90:	0.00068305	0.617956	18	0.93773
RP2	Negative	Cabazitaxel	IC50:	0.00027897	0.726623	6	-0.64499
			IC90:	0.00068564	0.837155	12	-0.74721
SMPD2	Negative	Cabazitaxel	IC50:	0.0025791	0.810049	59	-0.54197
			IC90:	0.00049137	0.837155	9	-0.5762
TCEA2	Positive	Cabazitaxel	IC50:	0.0019373	0.49995	71	0.53082
			IC90:	0.0014109	0.617956	41	0.58067
ZNF585B	Negative	Cabazitaxel	IC50:	0.00011941	0.570545	4	-0.35592
			IC90:	0.00070015	0.837155	13	-0.38244

Supplementary Table S3. Candidate genes. The name of each selected candidate gene is listed along with CRISPR/Cas9 screen type and MAGeCK information. FDR: false discovery rate. LFC: log fold change. *: *DRG1* was selected as a candidate gene in both the docetaxel and cabazitaxel screen.

Gene	sgRNA sequence (5'-3')	KO population name	KO population indel frequency (%)	KO single cell clone name	KO single cell clone indel frequency (%)
BCL2L13	GCAGAGTACATCATTCAGCA	C4_pop_BCL2L13	69.0	-	-
CEP135	AGCTCAAAAATTTAGCCATG	C4_pop_CEP135	73.0	-	-
E2F4	GATGTCCTCATGAGTGACGT	C4_pop_E2F4	72.0	• C4_scc_E2F4_1<br>• C4_scc_E2F4_2	• 85<br>• 83
IP6K2	AATAGCATATCCATTGAAAG	C4_pop_IP6K2	66.8	• C4_scc_IP6K2_1<br>• C4_scc_IP6K2_2<br>• C4_scc_IP6K2_3	• 95<br>• 93<br>• 97
KDM6A	CCAACTATCTAACTCCACTC	C4_pop_KDM6A	72.0	-	-
SMS	AAATATTCTCATCCTTAGTG	C4_pop_SMS		• C4_scc_SMS_1<br>• C4_scc_SMS_2	• 87<br>• 92
XPO4	GATGTCGTTGTAACTGACCA	C4_pop_XPO4	86.5	• C4_scc_XPO4_1<br>• C4_scc_XPO4_2<br>• C4_scc_XPO4_3	• 93<br>• 97<br>• 88
DRG1	GAGCCTTAAGCAGCCCTAAG	C4_pop_DRG1	60.0	• C4_scc_DRG1_1	• 84
LMO7	AAGATGATATGTCGTATCGA	C4_pop_LMO7	73.0	• C4_scc_LMO7_1	• 89
NCOA2	GGGAGGATTCATATTAACTG	C4_pop_NCOA2	81.0	-	-
ZNF268	CCACACTGAATACACCCAT	C4_pop_ZNF268	67.0	-	-
ARHGAP11B	AAGAACATATTCATACCGAA	C4_pop_ARHGAP11B	51.7	• C4_scc_ARHGAP11B_1<br>• C4_scc_ARHGAP11B_2	• 84.2<br>• 64.8
FKBP5	CAATTCCAATTGGAATGTCG	C4_pop_FKBP5	64.7	• C4_scc_FKBP5_1<br>• C4_scc_FKBP5_2	• 85.8<br>• 57.9
FRYL	AGTTTGATTGGTGACCGACG	C4_pop_FRYL	80.9	• C4_scc_FRYL_1<br>• C4_scc_FRYL_2<br>• C4_scc_FRYL_3	• 93.9<br>• 83.8<br>• 68.9
PRKAB1	GTGGACGCACGACCCTTCCG	C4_pop_PRKAB1	81.5	• C4_scc_PRKAB1_1<br>• C4_scc_PRKAB1_2<br>• C4_scc_PRKAB1_3	• 95.6<br>• 95.2<br>• 91.9
RP2	AGCATTGTTCCAATATCCCG	C4_pop_RP2	77.6	• C4_scc_RP2_1<br>• C4_scc_RP2_2<br>• C4_scc_RP2_3	• 93.6<br>• 96.7<br>• 93.0
SMPD2	ACAGGCTTTGACCCTCACAG	C4_pop_SMPD2	51.5	-	-

TCEA2	GGCCTCTGAGGCATCCCTCG	C4_pop_TCEA2	40.0	-	-
ZNF585B	ATGTATGTATTGAATGTGGG	C4_pop_ZNF585B	77.2	• C4_scc_ZNF585B_1<br>• C4_scc_ZNF585B_2<br>• C4_scc_ZNF585B_3	• 91.3<br>• 92.2<br>• 96.8

Supplementary Table S4. Overview of knockout cell line populations and single cell clones. KO: knockout.

**Primer sequence (5'-3'):**	ACTATCATATGCTTACCGTAAC

Supplementary Table S5. Primer for validation of correct sgRNA insert. Sequence of the primer used to validate correct sgRNA insert in the PX458 plasmid during cloning of constructs for generation of individual knockout cell lines.

**Gene**		**Sanger sequencing primer**	**RT-qPCR primer**
BCL2L13	Fwd:	GCCTCTGGTTTTGCTACGAC	AGGGCCAGGGGTTCAACTA
	Rev:	CCGGGTAACACAGCAAGAAC	CAGGCTTTCTGTGTGCCAAC
CEP135	Fwd:	GCCAGTGGTTTCCTTCCTGT	TGAACATTTGACATGTGTTAATCATCAG
	Rev:	ACCACAAACACATTCCCAGAAAT	TGGAAAGTCGGTGCTGATAGTCATC
E2F4	Fwd:	TCAAGGCAGAGATCGAGGAG	CAAGGTGTGGGTGCAGCAGAG
	Rev:	CTTCTGCCCATTGAGACCCT	CTGGAGCAAATCTTCAGGTGGTG
IP6K2	Fwd:	TCTCCTCTTTACGGTGCTGA	GAGATGCGCAAATTCACTCC
	Rev:	TTCTTTCTGCTCCTCACCCC	TTCAGATTTCTTTAGCCACTCAAA
KDM6A	Fwd:	GGAAGTGGAAGTAATGGAAACG	ATTACAGCATTTGGAACAGCTC
	Rev:	AACATTGTAGTGCCACAAAATTC	GTGTGCCTGCTTGTTTCAGG
SMS	Fwd:	GCGCCTGGTTGAATATGACATA	CTCGGCGCCAAAGCTGAT
	Rev:	AATATGCCTCCGTCTCCACC	GCCACACGTTTTTCGCATGT
XPO4	Fwd:	ACCACCAACCAACCTGTAAC	CGTGAGGAGGAAGAAATAAGTGA
	Rev:	CCAGTTTTCAAATGCCTGTTGT	AGTGGCTGAAGAACCTCTGTC
DRG1	Fwd:	TCCTCTCAAAGCTGGGGGAA	TAATTCGAAGTACCGCGCCT
	Rev:	ATTTGCCTCTGAAACCGGCA	CGGGCCACTGCAATGACTT
LMO7	Fwd:	GCAGCACTGGGTTTCTAAGG	TCGAGGTCATTGACAAGCTG
	Rev:	GGCTGTCTACTTTTGTTGGGTAA	GCTGACGCAAGTTTTGAACA
NCOA2	Fwd:	CTGCTTGTCCAGTTGTACCT	AGAAGGAGAAGATTTGCAGTCCT
	Rev:	GCTCCCTCAATGCACTTCAG	CATGTCCTGACCTGGGTTCC
ZNF268	Fwd:	TCAGTTTCCATTCACAGCTTGT	AGTCCTGGGGACCTTTGTCA
	Rev:	AAAAGCCTTTCCACAGTCGG	ATGACTTGCTGCTGAAGGCT
ARHGAP11B	Fwd:	AGATGGGCCTTGGAGAAGAA	TTGGAAGACTTGGTGGCGAA
	Rev:	TGCCACCACACTCAGCTATA	GTACACGCCCTTCTTTTCTGC
FKBP5	Fwd:	GAAATGAGAAAGGGGAAATGG	TCTTGGCAAAGGCCAAGTCA

	Rev:	GCAATACTGCAGGCAAAACA	GGCAGCCTGCTCCAATTTTT
FRYL	Fwd:	TCTCAGCAAGTAGAGCCTCC	CAGGAGAAGATGCACAGGGAT
	Rev:	GTTGAGAAGCCTGAGAGCCA	TTTACTGAGGAGGTGCACGG
PRKAB1	Fwd:	TCAGGAAACTGTGTCGCACT	TCCGAGGAAATCAAGGCACC
	Rev:	CTATGGGCTGGGAAGAGACA	GCCAAGCTGGCTGGTTACTA
RP2	Fwd:	TTTCCGGAATTGCAGAGATT	GCCTGCCAACAATTTCGTGT
	Rev:	TGAGGGTTGCCTGTATTTCC	CCAAGGCAATAACAGGACCTTTG
SMPD2	Fwd:	ATGGGTGGAAAGTGGGGTAG	GCCCAGTTCATCCACCACA
	Rev:	GAGTCAAGAGTGGAAGGGCT	TAGAATGCACCTGCCCACAG
TCEA2	Fwd:	CAGGACTGCAGAGATGGACA	AGTCGGGATGTCTGTCAACG
	Rev:	GGACACAGACCCACATCTCA	GGTCTGCACCTGTGTGTAGG
ZNF585B	Fwd:	TGGAAATGGCTTGAAGCATAA	CAGCTGCCCAGGAGAGAAAT
	Rev:	GGAAACCTTTCCCACATTCA	CTGGATGAAGGCCTGCCC
GAPDH	Fwd:	-	ATGGGGAAGGTGAAGGTCGG
	Rev:	-	GACGGTGCCATGGAATTTGC
UBC	Fwd:	-	GATTTGGGTGCGGGTTCTT
	Rev:	-	TGCCTTGACATTCTCGATGGT

Supplementary Table S6. Sanger sequencing primers and RT-qPCR primers used to validate knockout of candidate genes.
